# Editorial: Scaling-up equitable nutritional care for girls and women in South Asia

**DOI:** 10.3389/fnut.2025.1587731

**Published:** 2025-04-16

**Authors:** Neena Bhatia, Manisha Nair, Charu Arora, Divya Tripathi, Zivai Murira, Vani Sethi

**Affiliations:** ^1^Department of Food & Nutrition and Food Technology, Lady Irwin College New Delhi, New Delhi, India; ^2^Nuffield Department of Population Health, University of Oxford, Oxford, United Kingdom; ^3^Srimanta Sankaradeva University of Health Sciences, Guwahati, Assam, India; ^4^Maternal and Fertility Nutrition Specialist, New Delhi, India; ^5^Department of Nutrition and Dietetics, Manav Rachna International Institute of Research and Studies, Faridabad, New Delhi, India; ^6^Nutrition Section, UNICEF Regional Office for South Asia, Kathmandu, Nepal

**Keywords:** South Asia, women's nutrition, adolescent nutrition, malnutrition, triple burden

The right to good nutrition is every woman's right to support her wellbeing and key to reducing the annual burden of low birth weight (~8 million) in South Asia. This Research Topic of 13 selected papers from a call for papers on “***Scaling-up equitable nutritional care for girls and women in South Asia*”** in July 2023 draws attention to the evidence on the status, policy and program action and provides recommendations. These articles are published across Frontiers in Nutrition Epidemiology and Frontiers in Public Health and provide five messages described below.

## Urgent action is needed to tackle the triple burden of maternal malnutrition

In South Asia, one in five women (22%) are underweight, one in five (20%) suffer from obesity and anemia in girls and women remains a persistent problem (49%). Malnutrition among married nulliparous women <24 years is particularly concerning. Kumar et al. using DHS data between 2010 and 2022 from six countries (Bangladesh, India, Maldives, Nepal, Pakistan, and Sri Lanka), showed that an estimated one in four nulliparous women 15–24 years were underweight or overweight in pooled analysis, with substantial variation between countries and declining trends of underweight and increasing trends of obesity and those from rural or urban poorer households or with lower education more likely to be underweight or low stature. Panchal et al. studied these patterns on refugees and revealed similar results, highlighting a need to deliver double duty actions based on geographic and demographic characteristics.

## Strong nutrition policy frameworks, but implementation gaps persist

Despite strong policy intents and frameworks, effectiveness of nutrition programs is limited due to gaps in essential nutrition supplies, training, human resources, monitoring and research to support programmes and budgets. The article by Sethi and Murira urges that effective nutrition policies and programmes in South Asia should address systems bottlenecks to deliver five essential multi-sector actions—(i) access to fortified and nutritious foods, (ii) micronutrient supplementation, (iii) nutrition information and counseling, (iv) infection prevention, and (v) services for women with nutritional risks (thin, short, overweight/obese, young and anemic) through co-operation between health, nutrition, agriculture, education, and social protection sectors. Sethi and Murira also urge to tap missed opportunities for integrating key maternal services into the same platforms that treat children, reaching women in the preconception period through antenatal nutrition platforms as well as differentiated strategies e.g., to reach the most marginalized communities.

## Women's groups show promise but require sustained investments

Women's groups can play a key role in transforming the approach to nutrition programs from one oriented principally around service delivery to one based on women's rights. Seven key pathways for impact have been highlighted in the article by Shrivastava et al. for engaging with women's groups and organizations that are working on women's rights which do not necessarily include nutrition rights. These pathways include income generation, agriculture, health education, rights advocacy, food access, cash transfers, and integrated service delivery. Mondal et al. highlight that while self-help groups have a positive influence on the health and eating habits of women and children, they are found to be less effective in changing nutritional outcomes for women and children or neonatal mortality (1). The literature review by Verma et al. examines the role of women led self help and support groups in improving health and nutrition outcomes in India, Bangladesh, and Vietnam observed that—while the core focus of self-help group initiatives has tended to be rural economic empowerment, ensuring sustainability is a common challenge faced by such initiatives and recommend the need to allow time and sufficient resources for group maturation, to prevent group dissolution, and to maintain quality by ensuring facilitators receive refresher training at regular intervals.

## Promising approaches from Bangladesh, India, Nepal, and Pakistan

- **Bangladesh**: An intervention by Abdulloeva et al. in rural Bangladesh found that integrating child growth monitoring and promotion services into the existing child immunization programme, improving local government accountability, implementing unified health records, and expanding integrated maternal and child health services resulted in enhanced growth monitoring and maternal nutrition services.
- **India**: Dhabhai et al. demonstrated two thirds of women who have not gained a healthy amount of weight during their pregnancy can achieve adequate weight gain in the subsequent four weeks when given a comprehensive package of nutrition services. The package included supplementation with high-quality, protein-rich food alongside close monitoring for adherence, delivering nutritional counseling, and referring to health facilities where necessary. Hazra et al. showed that initial grants and seeds to households for growing kitchen gardens can act as a seasonal source of vegetables for the household and may help improve dietary diversity.
- **Nepal:** Cunningham et al. demonstrate that delivering social and behavior change communication through at least two channels at the same time can significantly improve maternal and child nutrition practices in disadvantaged social and economic population groups. Saville et al. showed that virtual counseling can also improve awareness and consumption of iron-rich diets in pregnant women.
- **Pakistan**: Samnani et al. develop a Nutrition Friendly School Initiative which involved developing school policies, engaging with and educating parents and established pathways for assessing children's nutritional status periodically. Naz et al., showed that community midwife led intra-venous iron therapy following prescription from consultant obstetricians reduced moderate anemia in a cohort of pregnant women in Karachi.

Together these papers provide following recommendations for accelerating progress to tackle the triple burden of malnutrition in women:

- **Develop and update multi-sectoral plans**: create clear targets and allocate budgets for women's nutrition services- before, during and beyond pregnancy, including nutrition risk assessments, monitoring and counseling, macro and micronutrient supplementation when required and prevention and treatment of infections with special care packages for those at risk of all forms of malnutrition. Investments will be needed from multiple sectors, especially education, health, social protection and food systems. Investments to protect women from nutrient-poor and unhealthy ultra-processed foods and beverages and to account for the differential strategies for marginalized communities needs to be made on priority. This will also entail updated service delivery intervention packages and toolkits to ensure comprehensiveness and alignment with global guidelines/recommendations, strategies to address system bottlenecks and implement a minimum nutrition package for women before, between and beyond pregnancies.
- **Women as agents of change**: leveraging women's movements and coalitions will enhance the visibility of women's nutrition rights within the broader women's rights agenda. Women's groups whilst can support peer-to-peer counseling can also play a critical role in addressing harmful gender and social norms that underlie maternal malnutrition and especially can promote those that work toward keeping girls in school, delay age at marriage. Additionally, nutrition social enterprises can offer dual opportunities for women groups for improving both nutrition and livelihoods.
- **Target inequities**: this would require being intentional in scaling up efforts to address social and geographical nutrition inequalities as revealed by subnational data to identify and close service delivery gaps for marginalized groups, particularly malnourished adolescent girls and women who are at economic, social or geographic disadvantage, particularly in humanitarian settings.
- **Invest in knowledge:** strengthen survey data systems to track trends in malnutrition, rigorously evaluate new interventions to support scale-up, promote academic-government collaboration for evidence-based policymaking, and encourage knowledge exchange within and between South Asian countries. Promoting effective use and dissemination of data will further ensure that progress in implementation of strategies are both accessible and accountable to the communities they aim to serve.

We are hopeful this Research Topic will trigger conversations for accelerating scaling-up efforts in improving nutrition of adolescent girls and women in South Asia, through convergent multi-sector nutrition solutions.
